# Effects of Fermented Soybean Meal Supplementation on Growth, Carcass Quality, and Intestinal Morphology in Ross 308 and Indian River Broilers

**DOI:** 10.3390/ani15182659

**Published:** 2025-09-11

**Authors:** Mohammad D. Obeidat, Sadeh Q. Alzoubi, Basheer M. Nusairat, Belal S. Obeidat, David G. Riley

**Affiliations:** 1 Department of Animal Production, Faculty of Agriculture, Jordan University of Science and Technology (JUST), Irbid 22110, Jordan; sadehalzoubi@gmail.com (S.Q.A.); bmnusairat@just.edu.jo (B.M.N.); bobeidat@just.edu.jo (B.S.O.); 2 Department of Animal Science, College of Agriculture and life Sciences, Texas A&M University, College Station, TX 77843, USA; david.riley@ag.tamu.edu

**Keywords:** fermented soybean meal, broiler strains, growth performance, intestinal morphology

## Abstract

A total of 700 broiler chicks (350 Indian River and 350 Ross 308) were obtained from a local hatchery. Birds were randomly allocated in a completely randomized design by strain into two dietary groups: a control group (corn–soybean meal) and a treatment group in which 7.5% of soybean meal (SBM) was replaced with fermented soybean meal (FSBM). Each group included 175 birds, distributed across seven replicates (pens) with 25 birds per pen. Birds were weighed at the start of the trial and at the end of each rearing phase (starter (1–14 days of age), and grower phases (15–35 days of age)). Mortality was monitored daily throughout the experiment. Weekly feed intake was recorded for each pen. On day 35, 14 birds were randomly selected from each treatment–strain combination for carcass and meat quality evaluation. Data were analyzed with linear models; strain, diet, and their interaction were the investigated effects. No strain–diet interaction was detected for growth performance, carcass and meat quality, and intestinal morphology traits (*p* > 0.05). Significant interaction effect on crude protein digestibility is found (*p* < 0.001). The FSBM improved crude protein digestibility for the Indian River broiler strain, while the opposite occurred in the Ross 308 strain. Fermented soybean meal supplementation did not significantly affect body weight gain, feed conversion ratio (FCR), or feed intake during the study period (*p* > 0.05). The Ross strain exhibited a higher dressing percentage (*p* = 0.04) and greater cooking loss (*p* = 0.01), whereas the Indian River strain showed higher abdominal fat percentage (*p* = 0.04) and shear force (*p* = 0.003). The dressing percentage, pH, color parameters, cooking loss, water-holding capacity, and breast fillet yield for FSBM and control groups did not differ (*p* > 0.05). Ileum villus length and width were significantly higher (*p* < 0.05) in the FSBM group compared to the control group. In conclusion, Fermented soybean meal can enhance intestinal morphology and crude protein digestibility in a strain-specific manner; however, its impact on growth and carcass attributes is limited at this inclusion level.

## Simple Summary

Fermentation improves soybean meal by enhancing its nutritional value while decreasing antinutritional components. This study investigated how adding fermented soybean meal (FSBM) to the starter diets of two broiler chicken breeds, Ross 308 and Indian River, affected their growth, intestinal health, and carcass characteristics. The important findings were that, while the FSBM had no significant effect on overall growth or carcass quality, it did improve intestinal morphology by increasing villus length and width. Furthermore, FSBM improved crude protein digestibility, particularly in the Indian River strain.

## 1. Introduction

The first two weeks post-hatching are critical for chicks, during which rapid growth and organ development occur [1]. During this period, a chick’s body weight doubles multiple times, supported by the rapid development of organs such as the heart, liver, and digestive tract [2]. Although numerous nutrients are essential for optimal growth and health, the chick’s underdeveloped digestive tract limits their absorption [3]. Enhancing nutrient, especially protein uptake during the early days of life can significantly improve overall performance [4]. Therefore, specially formulated starter feeds are essential to compensate for the immature digestive capacity of chicks. The diet of broiler chickens is typically composed of maize and soybean meal (SBM). Despite its high caloric and protein content, this diet also contains considerable quantities of non-starch polysaccharides, which contribute to the formation of a viscous environment in the intestine and reduce birds’ performance [5]. Soybean meal is the most commonly used protein source in the poultry feed industry. However, its use is limited by variability in nutritional quality and the presence of anti-nutritional factors (ANFs) [6]. Fermentation, a traditional method of food processing, enhances the nutritional and functional properties of feed ingredients [7]. It is widely employed to increase nutrient bioavailability and reduce ANFs in SBM [8]. Fermented feed has also been shown to boost circulating immunoglobulin levels and enhance intestinal immunity in broiler chickens [9]. Fermentation is an economical and effective method to improve the nutritional value of soybeans by reducing ANFs while preserving high-quality protein [7]. Fermentation effectively reduces soybean ANFs such as trypsin inhibitors, phytates, galactooligosaccharides, and lectins [10]. Compared to conventional SBM, fermented soybean meal (FSBM) contains higher levels of small peptides, fats, minerals, and vitamins [11]. Overall, fermentation enhances nutrient digestibility and absorption, supporting improved poultry health and performance [12]. Due to its relatively high cost, FSBM is generally included only in pre-starter broiler diets [13]. HP AviStart (Hamlet Protein, Horsens, Denmark) is a specialized FSBM product with minimal ANFs, which helps reduce the negative effects on nutrient digestibility and absorption [14]. Its high digestibility ensures optimal delivery of essential amino acids to chicks [10].

This study hypothesizes that inclusion of FSBM during the starter phase will improve gut morphology and protein digestibility, thereby enhancing growth and carcass traits in broilers. We sought to evaluate these effects across two commercial strains to see if the dietary supplement’s benefits are universal or contingent upon the bird’s genetic composition.

## 2. Materials and Methods

### 2.1. Animal Ethics and Housing Conditions

The experiment was conducted in the Poultry House at Jordan University of Science and Technology (JUST). All animal care protocols and experimental procedures were approved by the Animal Care and Use Committee at JUST (Approval #: 14/3/10/520). A total of 700 mixed-sex one-day-old chicks (350 Ross 308 and 350 Indian River) were obtained from a commercial hatchery, weighed, and randomly assigned to four treatment–strain groups, each with seven replicate pens (2 × 1.5 m) of 25 birds, in an open-sided, naturally ventilated house with 23 h light and 1 h dark. Feed and water were provided ad libitum. Temperature was kept at 32 °C on the first day and reduced gradually using cooling fans to reach 21 °C at day 35. The relative humidity inside the house ranged from 45 to 50. Light intensity ranged from 20 to 30 lx for the first 5 days and then reduced to 10 lx until day 35. All pens were covered with new wood shavings as bedding litter. The starter (day 1 to 14) and grower (day 15 to 35) diets were formulated to meet or exceed broiler nutrient requirements, adhering to both the National Research Council (NRC) guidelines [15] and the specific recommendations from the broiler strain producer. Diets were mixed weekly and sampled at mixing to ensure consistency in chemical composition. Birds were vaccinated against Infectious Bronchitis virus (IBV), Newcastle disease virus (NDV), and avian influenza.

### 2.2. Experimental Design and Diets

The four diets included two control corn–SBM-based formulations and two treatment diets where 7.5% of SBM was replaced by fermented soybean meal (FSBM; HP-AVI^®^) during the starter phase. FSBM was produced by co-fermenting soybeans with yeast. Table 1 presents the nutritional components profile of the FSBM used.

All experimental diets were formulated to ensure they delivered consistent levels of metabolizable energy (ME) and crude protein (CP) within each specific feeding phase. Crude protein, essential amino acids (lysine, methionine, threonine), minerals (calcium, available phosphorus), and vitamins were balanced across all diets. The ingredient composition and analyzed nutrient content of the diets are detailed in Table 2.

### 2.3. Growth Performance Assessment

At the beginning of each week, a predetermined amount of the feed (following the strains management guidelines) was weighed and distributed to each replicate within each treatment. The remaining feed was weighed and deducted from the stated quantity to determine the overall feed intake for the week. Birds were weighed at the end of each rearing phase—starter (days 1 to 14) and grower (days 15 to 35) at 8.30 A.M. Feed consumption per replicate was calculated by subtracting the leftover feed from the initial quantity provided. Average daily gain (ADG), average daily feed intake (ADFI), and feed conversion ratio (FCR) were calculated for each phase and for the total trial period (days 1–35).

Weekly body weight gain and FCR were corrected for mortality and calculated throughout the experimental period using the following formulas:

BWG = (Final weight − Initial weight)/Number of days

FCR = Average feed intake (g)/Average body weight gain (g)

### 2.4. Digestibility Trial

Titanium dioxide (TiO_2_) in diets and ileal samples was determined using the method of Myers et al. [16]. Titanium dioxide was included at 2% in the diets as an indigestible marker to assess Apparent Total Tract Digestibility (ATTD). The TiO_2_-containing diets were fed for three consecutive days (days 15–18). TiO_2_ was manually mixed to ensure uniform distribution in the feed. Briefly, the weighted TiO_2_ was thoroughly mixed with just a small quantity of finely crushed corn to create the TiO_2_ concentrate. To ensure thorough dispersion, this concentrate was then gradually added to the larger diet batches and manually (hand) mixed for 30 min every batch. Then, three sub-samples were taken from various locations within each mixed batch to confirm homogeneity, and the content of TiO_2_ was then measured. Its use as an external marker for digestibility calculations was validated by the analytical data, which showed a low coefficient of variation (<10%) among samples within each diet. On day four, birds were anesthetized using chloroform, after which the body cavity was opened, and the ileum was excised and flushed to collect digesta. Collected excreta samples were stored at −20 °C.

Following freeze-drying, crude fat content was determined in both feed and ileal digesta for digestibility calculations. Samples were digested using the wet digestion method, then treated with H_2_O_2_ to produce an orange/yellow color, which was measured at 410 nm using a spectrophotometer (SPECTRO UV-VIS DOUBLE BEAM MODEL SPECTRO UVD3000 Type, LABOMED Inc., Los Angeles, CA, USA) [16]. A standard curve was generated using a titanium dioxide stock solution (100 mg/75 mL). The analytical technique used was a modified spectrophotometric approach founded on the ideas presented by Myers et al. [16]. All reagents (sulfuric acid, hydrogen peroxide, and standards) were prepared from analytical-grade chemicals.

Excreta samples were oven-dried at 57 °C for 72 h and ground to pass through a 1 mm screen. Crude protein content was determined using the Kjeldahl method. Titanium dioxide was quantified after ashing the samples and treating the ash with boiling hydrochloric acid. The ATTD of dry matter and nitrogen was calculated using the indirect ratio method. Gross energy (GE) of feed and excreta was determined using a bomb calorimeter. Nutrient digestibility was calculated using the following formula [17]:

Digestibility (%) = 100 − [100 × (% marker in feed/% marker in feces) × (% nutrient in feces/% nutrient in feed)].(1)

### 2.5. Carcass and Meat Quality Measurements

On day 35, after a 12 h fast, final live weights were recorded, and 14 birds were randomly selected from each treatment–strain group for carcass and meat quality evaluation. The slaughtering procedure followed the method described by [18]. Carcasses were then weighed and dissected into five standard portions: breast, legs, wings, neck, and back. Breast cuts were excised from carcasses by cutting them into forequarters and leg quarters as described by Hudspeth et al. [19]. Cut weights were recorded, and dressing percentage was calculated as the ratio of carcass weight to live weight. Meat quality parameters were assessed on both right and left pectoralis major muscles. Measured traits included cooking loss (CL) and Warner–Bratzler shear force (SF) of cooked breast muscle. Color coordinates (L*, a*, b*), pH, and water-holding capacity (WHC) were also evaluated on uncooked meat samples. Liver and pancreas weights were recorded separately from 10 birds per treatment group. Entire breast samples from each carcass were sealed in labeled polyethylene trays, wrapped in wax paper, and stored at −32 °C.

Meat quality was assessed using 40 breast samples (10 per treatment group) obtained from slaughtered birds. Frozen breast samples were thawed overnight at 4 °C while still sealed in plastic bags. Samples were unwrapped, weighed, deboned, and the left and right pectoralis major muscles were separated for further analysis. The left pectoralis major muscle was used for meat quality measurements. Cooking loss and shear force were evaluated on cooked left pectoralis samples, while pH, WHC, and color were assessed using the uncooked right pectoralis major [20]. Briefly, the left pectoralis muscle was weighed and placed in poly-ethanol bags and cooked for 25 min at 85 °C, then the cooking loss was reported as the weight loss during cooking divided by sample weight and expressed as a percentage. Shear force was determined on 6 cores (20 × 13 × 13 mm) from the left pectoralis major muscle (caudal portion of the breast, perpendicular to muscle fiber) after being cooked for 90 min at 75 °C using a Warner–Bratzler shear force blade with the triangular slot cutting edge mounted to Salter model 235 (Warner–Bratzler meat shear, G-R manufacturing co. 1317 Colins LN, Manhattan, KS, 66502, USA) to determine the peak force (kg) when shearing the samples. Color coordinates were assessed using a colorimeter (12MM Aperture U 59730-30, Cole-parameter International, Accuracy Micro sensors, Inc. Pittsford, New York, NY, USA), calibrated using a standard white ceramic reference (CIE L* = 97.91, a* = −0.68, b* = 2.45) under a standard illuminate (illuminate D65). pH was determined in duplicate samples using a pH meter (pH spear, large screen, waterproof pH/temperature Tester, double injection, model 35634-40, Eurotech instrument, Kuala Lumpur, Malaysia). The pH meter was calibrated by measuring buffer solutions at pH 4.0 and 7.0 at ambient temperature.

### 2.6. Histological Analysis of Intestine

Intestinal tissue preparation followed the procedure outlined in jejunal samples (pieces of approximately 1 cm were taken from the middle part of the jejunum), which were cut into 5 mm segments, fixed in formalin, dehydrated in graded alcohol using a Histokinette device 2000, and embedded in paraffin. Dehydrated tissues were embedded in paraffin at 75 °C using a paraffin dispenser and stored in cassettes. The paraffin blocks were cooled at room temperature and stored until sectioning. Using a microtome, frozen tissue cassettes embedded in paraffin were cut into 5 µm slices. The slices were then floated in a water bath at 47 °C, placed on slides, and allowed to air dry. Slides were initially dewaxed in xylene for four minutes before being rehydrated using ethanol concentrations ranging from 70% to 100% for PAS staining. After five minutes of periodic acid treatment, the tissues were washed with tap water and incubated for half an hour in Schiff’s reagent to allow the stain to absorb. Slides were dehydrated using ethanol concentrations ranging from 70% to 100% and then soaked in xylene after excess stain was removed with tap water. The slides were then taken out of the rack, cover slipped, mounted with DEPX, and left to dry at room temperature. Five slides per bird were stained using the periodic acid–Schiff (PAS) method. Morphometric parameters of the jejunal villi were measured under a compound microscope.

Villus height, villus width, and crypt depth were measured on each slide. A digital microscope connected to (Motic Image 2.0 multi-language, Motic China Group Co., Ltd., Xiamen, China) software was used to capture and analyze histological images. Image analysis was used to quantify villus length, width, and crypt depth for each treatment group. Villus height and crypt depth were measured from 5 villi per bird, and only intact and unbroken villi were considered.

### 2.7. Statistical Analysis

Data collected were analyzed using the PROC GLM procedure of SAS 9.4 (SAS Inst., Inc., Cary, NC, USA). Data were analyzed using two-way ANOVA, and Tukey post hoc comparison test (statistical significance was *p* < 0.05). Least squares means ± standard errors were reported. The effects of interest were strain and diet, and their interaction on all growth performance. For the carcass and meat quality traits, final body weight was included in the model as a covariate. Effect sizes were calculated using Partial Eta-squared (η^2^).

## 3. Results and Discussion

### 3.1. Growth Performance

#### 3.1.1. Feed Intake

Feed intake results are summarized in Table 3. No significant strain × treatment interaction was observed for feed intake for all experimental phases (*p* > 0.05). Feed intake during the trial was not significantly influenced (*p* = 0.875) by either strain or dietary treatment. This is consistent with [21], who found no effect of FSBM on feed intake during the grower and finisher phases. Similarly, Mathivanan et al. [22] observed no significant differences in feed intake until week 4; however, from weeks 5 to 6, birds fed 1.5% FSM showed significantly lower cumulative intake. Fermented feed reduced feed intake during the starter and grower phases, potentially contributing to slower growth [23]. This reduction may have resulted from decreased palatability or lower levels of essential nutrients, such as lysine [24]. In the current study, replacing SBM with FSBM had no significant effect (*p* = 0.421) on feed intake. Feed intake response to FSBM may vary depending on the microbial strains used during fermentation [25]. Furthermore, the inclusion level of FSBM could influence its effect on feed intake.

#### 3.1.2. Feed Conversion Ratio

Feed conversion ratio (FCR) values are presented in Table 4. No significant interaction between strain and treatment was observed for FCR during the rearing phases (*p* > 0.05). During days 1–14, the HP-AVI group had a significantly higher FCR than the control group (*p* = 0.002). No significant differences in FCR were observed among strains or treatments during the grower phase (days 15–35) (*p* = 0.954). Guo et al. [26] reported that FSBM levels between 2.5% and 7.5% did not significantly affect FCR during the grower and overall phases. Mathivanan et al. [22] observed that a 5% inclusion of FSBM fermented by *Aspergillus niger* reduced FCR during weeks 3 and 4. Discrepancies between studies may be due to variations in microbial strains, fermentation methods, or FSBM inclusion levels.

#### 3.1.3. Body Weight Gain

Body weight gain results are shown in Table 5. The interaction effect was not detected for either initial or final body weights or the body weight gains (*p* > 0.05). Ross chicks had a significantly lower initial body weight compared to Indian River chicks (*p* = 0.001). This finding aligns with Hameed et al. [27] who noted that chick weight is closely related to hatching egg weight and can be influenced by genetics, environmental conditions, and incubation management. Thus, the minor difference in initial body weight may be attributed to these factors. Final body weight did not differ significantly between strains (*p* = 0.092). The diets contained slightly lower protein levels than recommended, which may have limited the strains from fully expressing their genetic potential for growth. There was no significant effect of strain on body weight gain during either the starter or grower phases (*p* = 0.953). Although FSBM is known to enhance nutrient digestibility and growth performance, such effects were not observed in this study. The inclusion level (7.5%) may have been insufficient to produce measurable improvements in digestibility or growth. In contrast, Kim et al. [21] found no differences in body weight at day 7 between dietary treatments. However, chicks fed BF-SBM and YBF-SBM diets had significantly higher body weights than controls from day 14 onward (*p* < 0.05). Additionally, chicks fed pre-starter diets with BF-SBM, YBF-SBM, or LF-SBM2 exhibited greater weight gains during both the grower phase and the overall period. As previously noted, variations in microbial strains, fermentation protocols, and inclusion rates may explain the inconsistencies between studies.

### 3.2. Carcass and Meat Quality Traits

#### 3.2.1. Carcass Traits

Carcass characteristics are summarized in Table 6. No interaction effect was detected for all carcass traits (*p* > 0.05). The Ross strain exhibited a significantly higher dressing percentage than the Indian River strain (*p* = 0.04). Indian River birds had significantly higher abdominal fat pad percentages compared to Ross birds (*p* = 0.04). Birds fed the control diet had significantly higher breast yield percentages than those fed the FSBM diet (*p* = 0.01). Kim et al. [21] reported no significant effect of FSBM supplementation on breast or abdominal fat percentages. Similarly, Guo et al. [26] found no significant impact of FSBM supplementation on breast yield or abdominal fat content. However, Guo et al. [26,28] who evaluated three FSBM levels (2.5, 5, and 7.5%) noted that leg muscle yield was lower in the 2.5% and 5.0% FSBM groups compared to the control, while the 7.5% group showed intermediate values with no significant difference. Adding fermented soybean to a broiler’s diet may or may not impact carcass characteristics since the diet may already have an adequate amino acid profile, particularly for essential amino acids such as methionine and lysine, so adding extra highly digestible protein does not increase muscle growth. Furthermore, other factors, such as genetics, the total calorie content of the meal, or environmental conditions, may influence the bird’s carcass expansion in addition to protein availability. Furthermore, the results can be inconsistent and depend on the fermentation process applied, the amount of fermented soybean included in the diet, the broiler’s age, and genetic strain, all of which might influence how the bird consumes the additional nutrients [25,29].

#### 3.2.2. Meat Quality Traits

Meat quality traits are presented in Table 7. There was no significant interaction of strain and diet detected for any of the meat quality parameters (*p* > 0.05). Breast fillet percentage did not differ significantly between Ross and Indian River strains (*p* = 0.86). Ross birds had significantly higher cooking loss percentages (*p* = 0.01) and lower shear force values (*p* = 0.003) than Indian River birds. No other meat quality parameters were significantly affected by strain (*p* > 0.05). Breast fillet percentage was higher for the control group (*p* = 0.05) compared to the FSBM group. Shear force in the FSBM group was lower than the control (*p* = 0.04), while no other meat quality trait was affected by the inclusion of FSBM (*p* > 0.05).

Birds fed 7.5% FSBM had higher breast muscle pH compared to other treatment groups [26]. The authors also reported that the lowest pH values were in the control and 5.0% FSBM groups, with intermediate values in the 2.5% group (*p* < 0.05). Adding fermented soybean meal to a broiler’s feed may have no effect on meat quality parameters such as cooking loss, pH, water-holding capacity, or color, as these characteristics are mostly influenced by muscle physiology rather than nutrition. While fermentation enhances nutrient digestibility and intestinal health, it does not always result in a change in the final meat product. The effects vary greatly depending on the fermentation technique and inclusion amounts employed [25,26,30].

### 3.3. Intestinal Morphology and Crude Protein Digestibility

Intestinal morphology and crude protein digestibility are presented in Table 8. There was no strain × diet interaction in villi’s length, width, and crypt depth. However, a significant strain × diet interaction was observed for CPD% (*p* < 0.001). Fermented Soybean meal significantly improved CPD% in Indian River broilers, consistent with its ability to minimize anti-nutritional factors and the breakdown and reduction in large-sized proteins in FSBM [31]. Conversely, FSBM reduced CPD% in Ross broilers. Depending on the particular broiler strain, adding fermented soybean meal (FSBM) to their feed can have varied effects on their crude protein digestibility. These variations are mostly caused by the distinct genetic makeup and gut microbiota composition of each strain. While strains with less effective digestive systems can benefit greatly from the breakdown of anti-nutritional factors and improved nutrient availability, strains with innately efficient systems might not benefit from FSBM [26]. Similarly to this, each strain’s distinct microbial community influences how it reacts to the probiotic and prebiotic effects of FSBM, producing a range of outcomes in terms of protein digestion [26].

No significant differences between strains were observed in villi length, width, crypt depth, or crude protein digestibility (CPD%) (*p* > 0.05). Both the ileum villi length and width were significantly greater (*p* < 0.05) in the FSBM group compared to the control group. Crypt depth in the ileum did not differ significantly between treatment groups (*p* > 0.05). The enhanced intestinal morphology observed in this study may be attributed to the removal of trypsin inhibitors (TIs) and the breakdown of large proteins in SBM during fermentation. Trypsin inhibitors are known to inhibit trypsin and chymotrypsin activity, which can impair intestinal morphology [32]. Additionally, the improvement in villi structure may be linked to the degradation of antigenic compounds during fermentation. Previous studies have shown that fermentation breaks down large proteins into smaller peptides [33]. Our findings are consistent with those of [32], who demonstrated that fermentation with *Aspergillus oryzae* enhances SBM nutritional value and mitigates the adverse effects of ANFs on broilers.

Although fermented soybean diets have been shown to increase villi length and width (*p* < 0.05), this trend was only partially evident in the present study. This contrasts with findings by [22] who reported significant increases in villi length, morphology, and villi-to-crypt ratios in birds fed fermented diets. These improvements were likely due to differences in fermentation strains or inclusion levels.

## 4. Conclusions

Fermented soybean meal (FSBM) supplementation at a 7.5% inclusion level enhanced intestinal morphology by increasing villus length and width in both broiler strains studied, most likely by lowering antinutritional factors and breaking down proteins. This dietary additive improved crude protein digestibility in the Indian River broiler strain while having the reverse effect in the Ross 308 strain, indicating a strain-specific response. Despite these improvements in gut health and digestibility in one strain, the FSBM had no significant effect on overall growth performance (body weight increase, FCR) or most carcass and meat quality characteristics, such as cooking loss, water-holding capacity, and color. The study concluded that FSBM at this level provides digestive benefits for specific genetic strains but has little impact on overall productivity and carcass traits, emphasizing the need for additional economic evaluation.

## Figures and Tables

**Table 1:** Chemical composition and energy value of fermented soybean meal (FSBM).

**Chemical composition**	**HP Avistart**
Energy, kcal ME/kg	2287
Crude protein, %	55.50
Phosphorus, %	0.80
Calcium, %	0.25
**Anti-nutritional factors**	
Trypsin inhibitor (mg/g)	1.30
Beta-conglycinine (ppm)	2.00
Oligosaccharides (%)	1.00
- Stachyose	0.30
- Raffinose	0.40
Lectins	<1
Phytic acid	2.0

Hamlet Protein A/S Denmark Company (Horsens, Denmark) determined the nutritional composition of HP Avistart (HPA) and soybean meal (SBM).

**Table 2:** Starter and grower diet composition and nutrients analysis.

Ingredient (%)	DIET		
	Starter (1–14 d)		Grower (15–35 d)
	Treatment	Control	Control
**Corn**	55.87	54.2	55.15
**FSBM**	7.5	0.00	0.00
**Soybean meal 44%**	29.95	38.74	37.25
**Oil**	2.58	3.04	4.12
**DL-Methionine**	0.26	0.26	0.26
**Salt**	0.50	0.50	0.50
**L-Lysine**	0.09	0.108	0.08
**Limestone**	1.39	1.4	1.40
**Di-calcium phosphate**	1.65	1.54	1.56
**Choline Chloride 60%**	0.20	0.20	0.20
**L-Threonine**	0	0	0.008
**Vitamin and mineral premix ^1^**	0.01	0.01	0.01
**Total**	**100**	**100**	**100**
	**Calculate nutrition**		
**Metabolizable energy (kcal/kg)**	3000	3000	3050
**Crude protein**	21.97	21.70	21.00
**Crude fat**	5.09	5.36	6.39
**Crude fiber**	3.67	3.94	3.84
**Calcium**	0.90	0.90	0.90
**Available phosphate**	0.45	0.45	0.45
**Sodium**	0.20	0.20	0.20
** Dig Threonine**	0.83	0.82	0.80
**Dig Lysine**	1.26	1.26	1.2
**Dig Methionine**	0.55	0.55	0.54
**Dig cysteine**	0.34	0.34	0.33
**Dig Tryptophan**	0.27	0.26	0.25
**Dig Isoleucine**	0.98	0.98	0.95
**Dig Valine**	1.33	1.39	0.35
** Dig TSAA**	0.89	0.89	0.87
**Dig Arginine**	1.44	1.47	1.32

^1^ Vitamin premix supplied the following per kg of diet: 6614 IU vitamin A, 1984 IU vitamin D3, 33 IU vitamin E, 0.02 mg vitamin B12, 0.13 mg biotin, 1.98 mg 1.1 mg folic acid. Selenium premix provided 0.2 mg Se (as NaSeO).

**Table 3:** Means for bird strains and treatment on feed intake of broiler chicks raised to 35 days of age.

		Feed Intake (g)		
		1–14	15–35	1–35
**Strain**	**Ross**	553.1 ± 3.6	2339.4 ± 23.2	2892.5 ± 23.1
	**Indian River**	561.4 ± 3.6	2295.9 ± 23.2	2857.4 ± 23.1
	***p-value***	***0.12***	***0.19***	***0.29***
	**(*η*^2^)**	***0.0949***	***0.051***	***0.0414***
**Treatment**	**Control**	556.9 ± 3.6	2290.6 ± 23.2	2847.5 ± 23.1
	**HP-AVI**	557.7 ± 3.6	2344.7 ± 23.2	2902.4 ± 23.1
	***p-value***	***0.88***	***0.11***	***0.12***
	**(*η*^2^)**	***0.0007***	***0.101***	***0.1003***
**Interaction**	***Strain × TRT***	***0.79***	***0.24***	***0.38***
	**(*η*^2^)**	***0.0133***	***0.0140***	***0.0146***

Effect sizes are calculated using Partial Eta-square (*η*^2^).

**Table 4:** Means for bird strain and treatment on the feed conversion ratio (FCR) of broiler chicks raised to 35 days of age.

		FCR (g:g)		
		1–14	15–35	1–35
**Strain**	**Ross**	1.15 ± 0.01	1.55 ± 0.02	1.5 ± 0.01
	**Indian River**	1.14 ± 0.01	1.54 ± 0.02	1.5 ± 0.01
	***p-value***	***0.27***	***0.69***	***0.67***
	**(*η*^2^)**	***0.0343***	***0.023***	***0.0067***
**Treatment**	**Control**	1.13 ± 0.01 ^b^	1.53 ± 0.02	1.14 ± 0.01
	**HP-AVI**	1.16 ± 0.01 ^a^	1.56 ± 0.02	1.15 ± 0.01
	***p-value***	***0.002***	***0.19***	***0.11***
	**(*η*^2^)**	***0.3085***	***0.112***	***0.1021***
**Interaction**	***Strain × TRT***	***0.76***	***0.19***	***0.57***
	**(*η*^2^)**	***0.0191***	***0.0292***	***0.0138***

^a,b^ Means in a column within each variable with different superscripts differ significantly (*p* ≤ 0.05); Effect sizes are calculated using Partial Eta-square (*η*^2^).

**Table 5:** Means for bird strain and treatment on body weight gain (BWG) of broiler chicks raised to 35 days of age.

			BWG (g)			
		Initial BW (g)	1–14	15–35	1–35	Final BW (g)
**Strain**	**Ross**	38.7 ± 0.27 ^b^	477.6 ± 4.4	1510.5 ± 11.8	1988.0 ± 12.9	2026.8 ± 12.9
	**Indian River**	40.3 ± 0.27 ^a^	487.8 ± 4.4	1491.2 ± 11.8	1979.1 ± 12.9	2019.9 ± 12.9
	***p-value***	***0.001***	***0.1***	***0.24***	***0.63***	***0.69***
	**(*η*^2^)**	***0.490***	***0.0896***	***0.071***	***0.0494***	***0.006***
**Treatment**	**Control**	39.3 ± 0.20	487.9 ± 4.4	1499.3 ± 11.8	1987.2 ± 12.9	2026.5 ± 12.9
	**HP-AVI**	39.8 ± 0.20	477.5 ± 4.4	1502.4 ± 11.8	1979.9 ± 12.9	2019.6 ± 12.9
	***p-value***	***0.12***	***0.12***	***0.86***	***0.69***	***0.71***
	**(*η*^2^)**	***0.0518***	***0.0911***	***0.042***	***0.0012***	***0.0052***
**Interaction**	***Strain× TRT***	***-***	***0.31***	***0.84***	***0.69***	***0.35***
	**(*η*^2^)**	***-***	***0.226***	***0.121***	***0.1097***	***0.1003***

^a,b^ Means in a column within each variable with different superscripts differ significantly (*p* ≤ 0.05); Effect sizes are calculated using Partial Eta-square (*η*^2^). Initial weight interaction effect was not calculated.

**Table 6:** Effect of bird strain and dietary fermented soybean meal on carcass cuts and abdominal fat percentages (percentages expressed as a part of cold carcass weight).

		Trait						
		Dressing%	Breast%	Leg%	Wings%	Neck%	Fat Pad%	Offal%
**Strain**	**Ross**	79.8 ± 0.6 ^a^	37.4 ± 0.33	26.6 ± 0.2	9.4 ± 0.1	7.0 ± 0.2	0.74 ± 0.1 ^b^	5.8 ± 0.1
	**Indian River**	78.1 ± 0.6 ^b^	36.9 ± 0.33	26.8 ± 0.2	9.5 ± 0.1	6.9 ± 0.2	0.93 ± 0.1 ^a^	5.8 ± 0.1
	***p-value***	***0.04***	***0.41***	***0.4***	***0.35***	***0.37***	***0.04***	***0.77***
	**(*η*^2^)**	***0.110***	***0.015***	***0.017***	***0.022***	***0.021***	***0.330***	***0.002***
**Treatment**	**Control**	78.9 ± 0.6	37.8 ± 0.31 ^a^	26.4 ± 0.2	9.4 ± 0.1	7.0 ± 0.2	0.81 ± 0.1	5.9 ± 0.1
	**HP-AVI**	78.9 ± 0.6	36.5 ± 0.31 ^b^	26.9 ± 0.2	9.5 ± 0.1	6.9 ± 0.2	0.85 ± 0.1	5.8 ± 0.1
	***p-value***	***0.97***	***0.01***	***0.08***	***0.29***	***0.71***	***0.64***	***0.21***
	**(*η*^2^)**	***0.006***	***0.158***	***0.075***	***0.029***	***0.004***	***0.005***	***0.007***
**Interaction**	***Strain× TRT***	***0.53***	***0.09***	***0.41***	***0.64***	***0.29***	***0.81***	***0.97***
	**(*η*^2^)**	***0.106***	***0.071***	***0.095***	***0.071***	***0.029***	***0.122***	***0.029***

^a,b^ Means in a column within each variable with different superscripts differ significantly (*p* ≤ 0.05). Effect sizes are calculated using Partial Eta-square (η^2^).

**Table 7:** Effect of bird strain and dietary fermented soybean meal on meat quality parameters.

		Trait							
		Quality Measures ^1^					Color Coordinates ^2^		
		Breast Fillet %	pH	CL%	WHC%	SF	L*	a*	b*
**Strain**	**Ross**	75.0 ± 0.6	5.9 ± 0.06	36.7 ± 0.4 ^a^	32.0 ± 0.6	3.1 ± 0.1 ^b^	38.4 ± 0.2	3.4 ± 0.03	21.0 ± 0.2
	**Indian River**	74.8 ± 0.6	5.9 ± 0.06	32.2 ± 0.4 ^b^	33.3 ± 0.6	3.8 ± 0.1 ^a^	38.5 ± 0.2	3.4 ± 0.03	20.7 ± 0.2
	***p-value***	***0.86***	***0.82***	***0.01***	***0.05***	***0.003***	***0.78***	***0.17***	***0.12***
	**(*η*^2^)**	***0.001***	***0.002***	***0.617***	***0.099***	***0.147***	***0.002***	***0.045***	***0.045***
**Treatment**	**Control**	75.7 ± 0.6 ^a^	5.9 ± 0.06	34.3 ± 0.4	32.9 ± 0.5	3.8 ± 0.1 ^a^	38.5 ± 0.2	3.4 ± 0.03	21.0 ± 0.2
	**HP-AVI**	74.1 ± 0.6 ^b^	5.9 ± 0.06	34.8 ± 0.4	32.6 ± 0.5	3.1 ± 0.1 ^b^	38.4 ± 0.2	3.4 ± 0.03	20.7 ± 0.2
	***p-value***	***0.05***	***0.27***	***0.31***	***0.5***	***0.04***	***0.76***	***0.54***	***0.07***
	**(*η*^2^)**	***0.093***	***0.033***	***0.011***	***0.001***	***0.328***	***0.004***	***0.009***	***0.062***
**Interaction**	***Strain × TRT***	***0.47***	***0.10***	***0.54***	***0.38***	***0.71***	***0.33***	***0.52***	***0.08***
	**(*η*^2^)**	***0.016***	***0.036***	***0.063***	***0.047***	***0.052***	***0.025***	***0.015***	***0.019***

^a,b^ Means in a column within each variable with different superscript differ significantly (*p* ≤ 0.05); ^1^ CL%: Cooking loss; WHC%: Water holding capacity; SF: Shear force, kg/cm^2^. ^2^ L*: Lightness; a*: Redness; b*: Yellowness. Effect sizes are calculated using Partial Eta-square (*η*^2^).

**Table 8:** Effect of bird strain and dietary fermented soybean meal on intestinal characteristics and crude protein digestibility (CPD%) of broilers at 5 weeks of age.

		Villi Length, µm	Villi Width, µm	Crypt Depth, µm	CPD, % ^1^
**Strain**	**Ross**	391.11 ± 10.220	45.67 ± 1.866	68.78 ± 2.176	84.59 ± 0.435
	**Indian River**	368.34 ± 9.662	42.72 ± 1.721	69.32 ± 2.217	84.46 ± 0.314
	***p-value***	***0.096***	***0.185***	***0.865***	***0.737***
	**(*η*^2^)**	***0.0217***	***0.0114***	***0.0003***	***0.0027***
**Treatment**	**Control**	359.23 ± 11.499 ^b^	37.41 ± 1.261 ^b^	69.89 ± 2.503	84.77 ± 0.375
	**HP-AVI**	400.22 ± 7.464 ^a^	50.98 ± 1.835 ^a^	68.22 ± 1.834	84.29 ± 0.371
	***p-value***	***0.003***	***<0.001***	***0.595***	***0.228***
	**(*η*^2^)**	***0.704***	***0.239***	***0.0024***	***0.0363***
**Interactions ^2^**	**Control *×* R**	379.32 ± 17.824	37.74 ± 1.698	69.77 ± 3.305	85.11 ± 0.185 ^a^
	**Control *×* I**	339.14 ± 13.876	37.07 ± 1.892	70.01 ± 3.816	83.47 ± 0.551 ^b^
	**HP-AVI *×* R**	402.89 ± 9.885	53.60 ± 2.636	67.80 ± 2.877	83.82 ± 0.483 ^b^
	**HP-AVI *×* I**	397.55 ± 11.335	48.36 ± 2.504	68.63 ± 2.324	85.72 ± 0.159 ^a^
	***p-value***	***0.55***	***0.12***	***0.91***	***<0.001***
	**(*η*^2^)**	***0.105***	***0.0257***	***0.003***	***0.531***

^a,b^ Means in a column within each variable with different superscripts differ significantly (*p* ≤ 0.05). ^1^ CPD% = crude protein digestibility. ^2^ Interactions, Control × R = Ross without FSBM, Control *×* I = Indian River without FSBM, HP-AV × R = Ross with FSBM, and HP-AVI × I = Indian River with FSBM. R = *Ross* and I = *Indian River*. Effect sizes are calculated using Partial Eta-square (*η*^2^).

## Data Availability

Data will be available upon request.
